# Complete Genome Sequence of Anaerostipes caccae Strain L1-92^T^, a Butyrate-Producing Bacterium Isolated from Human Feces

**DOI:** 10.1128/MRA.00056-21

**Published:** 2021-04-22

**Authors:** Kana Morinaga, Hiroyuki Kusada, Miho Watanabe, Hideyuki Tamaki

**Affiliations:** a Bioproduction Research Institute, National Institute of Advanced Industrial Science and Technology, Tsukuba, Ibaraki, Japan; b Graduate School of Life and Environmental Sciences, University of Tsukuba, Tsukuba, Ibaraki, Japan; University of Maryland School of Medicine

## Abstract

Anaerostipes caccae strain L1-92^T^ is a well-known butyrate-producing bacterium that has been isolated from human feces. In this announcement, we present the complete genome sequence of *A. caccae* strain L1-92^T^, which comprises 3,590,719 bp with a G+C content of 44.30%. The genome harbors 3,369 predicted protein-coding genes.

## ANNOUNCEMENT

The genus *Anaerostipes* is one of the most abundant bacterial taxa in the human intestinal microbiome (1, 2). *Anaerostipes* spp. are considered a key gut microbe associated with human health and disease, since they are capable of producing butyrate, known to have beneficial effects on intestinal functions (3). Here, we provide the complete genome sequence of the type species of the genus *Anaerostipes*, *A. caccae* strain L1-92, which is a human feces-derived, Gram-positive, butyrate-producing bacterium (4).

*A. caccae* strain L1-92^T^ (=DSM 14662^T^=JCM 13470^T^=NCIMB13811^T^) was obtained from the Japan Collection of Microorganisms (RIKEN BRC, Tsukuba, Japan). The strain was inoculated into anaerobically prepared Gifu anaerobic medium with headspace gas of N_2_/CO_2_ (80:20 [vol/vol]) and incubated at 37°C for 2 days. Cells were collected by centrifugation at 8,000 × *g* for 10 min. Genomic DNA was extracted using enzyme-based DNA extraction methods as described previously (5) with some modifications. In brief, lysozyme, achromopeptidase, and proteinase K were used to lyse cells, followed by genomic DNA purification using the phenol-chloroform method. *De novo* sequencing using the HiSeq system (Illumina, San Diego, CA, USA) and PacBio (Menlo Park, CA, USA) sequencing platform and hybrid assembly were conducted at Genewiz, Inc. (South Plainfield, NJ, USA). Illumina paired-end (2 × 150-bp) reads (6,962,694 reads) were generated; then, libraries with different indices were multiplexed and loaded onto an Illumina HiSeq instrument according to the manufacturer’s instructions. Default parameters were used for all software. Sequencing was carried out using a 2 × 150-bp paired-end configuration; image analysis and base calling were conducted using the HiSeq control software + OLB + GAPipeline v1.6 (Illumina) on the HiSeq instrument. In addition, long sequence reads (343,145 reads) were generated, and the SMRTbell library was prepared according to the manufacturer’s instructions. The library was sequenced and analyzed using the PacBio RS II platform and single-molecule real-time (SMRT) sequencing technology (which yields sequences with ≥99.999% high-quality data) (6). The PacBio reads were assembled using HGAP4 v4.0/Falcon v0.3 of WGS-Assembler v8.2 (7). The *N*_50_ value was 5,455 bp. The genome sequence was further corrected using the Illumina HiSeq and PacBio reads with Pilon v1.22 (8) and Quiver (9), respectively. The genome circularization was confirmed by identifying HiSeq reads that map to the beginning and end of the assembly. This yielded a complete genome sequence for *A. caccae* strain L1-92^T^. The resulting sequence was annotated using the NCBI Prokaryotic Genome Annotation Pipeline (PGAP) v5.0 (10). The completeness and contamination level of the genome sequence were assessed using CheckM v1.0.7 with the “lineage_wf” workflow (11).

The genome sequence of *A. caccae* strain L1-92^T^ is comprised of a circular chromosome of 3,590,719 bp with a 44.30% G+C content. The numbers of predicted coding sequences and rRNA and tRNA genes in the genome were 3,369, 12, and 57, respectively. CheckM estimated the genome completeness as 99.33% and the contamination rate as 4.03%. The complete genome sequence of *A. caccae* strain L1-92^T^ provides essential data for future taxonomic, comparative genomics, and metabolic analysis and for gaining deep insights into how this strain affects human health and disease.

### Data availability.

The complete genome sequence and annotations of *A. caccae* strain L1-92^T^ have been deposited at DDBJ/EMBL/GenBank under accession number AP023027. The genome sequence has also been submitted to the SRA under BioSample accession number SAMD00215733 and BioProject number PRJDB9542. The raw sequence data for strain L1-92^T^ were deposited under DRA accession numbers DRR259155 (Illumina) and DRR259156 (PacBio).
